# Fate of Pyrrolizidine Alkaloids in Soil: Insights from *Myosotis arvensis* L. and *Senecio vulgaris* L.

**DOI:** 10.3390/toxins17070335

**Published:** 2025-07-02

**Authors:** Ilva Nakurte, Gundars Skudriņš, Ieva Mežaka

**Affiliations:** Institute for Environmental Solutions, Lidlauks, Priekuļi Parish, LV 4126 Cēsis County, Latvia; gundars.skudrins@vri.lv (G.S.);

**Keywords:** soil contamination, pyrrolizidine alkaloids, leaching behavior, forget-me-not, ragwort

## Abstract

Pyrrolizidine alkaloids are plant-derived toxins with environmental persistence and the potential to contaminate soil, water, and adjacent crops. This study investigated the leaching behavior and environmental fate of PAs from two PA-producing weeds—*Myosotis arvensis* L. (Boraginaceae) and *Senecio vulgaris* L. (Asteraceae)—in two Latvian agricultural soils: sandy loam and loam. Hot- and cold-water plant extracts were applied to soil columns (10 cm and 20 cm), and leachates were analyzed over a 14-day period using QuEChERS purification and LC-HRMS detection. Leaching varied by plant species, extract type, and soil. *M. arvensis* showed significantly higher cumulative leaching (77–84% for cold, 65–71% for hot extracts), attributed to the higher solubility of N-oxides. In contrast, *S. vulgaris* extracts leached minimally (<0.84% from sandy loam) and were undetectable in loam. The presence of cyclic diester PAs in *S. vulgaris* and the higher cation exchange capacity of loam favored retention or degradation. PANO-to-PA conversion occurred in both soils, indicating redox activity. The fate of PAs was influenced by structural type (diesters showing higher persistence), extraction method (hot extraction releasing more pyrrolizidine alkaloids), and soil properties such as pH, organic matter, and cation exchange capacity, which affected sorption and mobility. These findings underscore the significance of soil composition in controlling PA mobility and associated environmental risks. Future research should focus on long-term PA persistence across diverse soil types and investigate crop uptake potential and microbial degradation pathways under field conditions.

## 1. Introduction

Alkaloids are a large group of naturally occurring nitrogen-containing compounds found in many plants. Among them, pyrrolizidine alkaloids (PAs) are notable for their toxicity and widespread presence [1,2,3]. These secondary metabolites serve ecological roles by deterring herbivores and insects [4], suppressing microbial activity in the rhizosphere [5], and inhibiting neighboring plant growth through allelopathic interactions [6,7]. Such functions support plant survival, particularly in competitive or disturbed environments. Despite these ecological roles, pyrrolizidine alkaloids (PAs) pose significant toxicological risks. In livestock, PA-contaminated forage can lead to liver damage [8]. In humans, PAs can enter the food chain through several pathways, including herbal teas, contaminated crops, and bee products such as honey, where they may cause hepatotoxic effects [2,9,10,11]. To manage these risks, the European Union has implemented stringent regulations on PAs in food, spices, and herbal products, including limits defined under Commission Regulation (EU) 2023/915 [12,13]. Contamination of herbal materials often occurs through the co-harvesting of PA-producing weeds [14,15]. However, PA content in environmental water, including drinking water, remains unregulated [16].

The toxicity of PAs varies depending on their chemical structure. Generally, cyclic diesters (CDEs) are the most toxic, followed by open-chain diesters (DEs) and monoesters (MEs) [9]. A relative potency (REP) model introduced by Merz and Schrenk in 2016 allows for the assessment of individual PAs based on their structural attributes, assigning REP values from 1.0 (most toxic) to 0.01 (least toxic) [3,11,17,18,19]. This model enables a more nuanced evaluation of PA-associated risks. PAs enter soil through multiple mechanisms: wash-off from living plants during precipitation [16,20], decomposition of PA-containing plant material [21], and direct root exudation [22,23]. These compounds have been detected in both soil and water environments. For example, in areas densely populated by *Senecio jacobaea* L., PA concentrations ranged from 3 to 1349 µg/kg in soil and 4 to 270 µg/L in surface water [20]. In tropical farming systems in Ghana, PAs from *Chromolaena odorata* residues leached into soil and were taken up by maize crops [21]. Recent studies have demonstrated that once PAs enter the soil, they degrade slowly, with limited but measurable biodegradation occurring over time [24]. Even when they are no longer detectable, PAs may have migrated further—into nearby streams, groundwater, or surface water systems [16,23,25]—reaching concentrations from a few nanograms per liter [26] to several hundred micrograms per liter [27]. Recent findings have also highlighted the presence of PAs such as jacobine N-oxide and senecionine N-oxide in surface waters, occasionally at concentrations that pose ecological risks [28]. Their transport through soil is largely determined by PA solubility and mobility, which vary with soil composition and moisture conditions. In agricultural areas, these compounds can leach into groundwater—particularly where PA-producing plants proliferate—extending the potential for human exposure [29,30]. Environmental variables strongly influence PA mobility. Rainfall enhances both vertical and lateral transport of PAs into water bodies, while temperature affects degradation rates [16]. Soil texture also impacts fate: clay soils bind PAs via charge interactions [31], while sandy soils facilitate desorption and transformation of N-oxides into free-base forms [28]. Studies in Switzerland have detected PA contamination in small streams, with detection frequencies between 36% and 87% and rain-induced peaks approaching 100 ng/L [25]. Although knowledge of PA behavior in the environment is growing, data on long-term persistence and soil-specific effects remain limited. This is particularly relevant given the wide distribution of PA-producing plants.

About 6000 plant species have been identified to contain PAs. They occur in many genera within the Boraginaceae family, in the tribes *Eupatorieae* and *Senecioneae* of the Asteracea family, in the Leguminosae family (*Crotalaria* genus), and to a lesser extent in some other plant families [32]. In agricultural fields, PA-producing species most frequently belong to Asteraceae and Boraginaceae [33]. For this study, we selected *Senecio vulgaris* (Asteraceae) and *Myosotis arvensis* (Boraginaceae) as model species.

Common groundsel, *S. vulgaris* L., is an annual plant with limited natural habitats such as coastal dunes, dry forests with disturbed soil, and forest clearings [34,35]. The species has adapted to ruderal and agricultural habitats [34], and the natural range of distribution extends from Europe to Asia and Northern Africa [36]. The weed has spread worldwide and now is invasive in Australia, as well as in South and North America [37]. Due to its widespread distribution and high risks of contaminating food products, it is of particular concern in Central Europe [14]. The *Senecio* genus has been extensively studied regarding PA biosynthesis, composition, and content [4,38,39]. *Senecio* species are notable for their high PA concentrations, often producing some of the most toxic variants. Their widespread global distribution has further contributed to their research prominence. Studies conducted in Germany [40], the Czech Republic [41], Spain [39], and the United States [42] have confirmed the presence of PAs in *S. vulgaris* plants. These studies identified various PAs, including jacoline, seneciphylline, spartiodine, integerrimine, senecivernine, senecionine, erucifoline, jacozine, jacobine, retrorsine, senkirkine, otosenine, and jakonine, along with their corresponding N-oxide forms.

Field forget-me-not, *M. arvensis* L. Hill (Boraginaceae), is a weed in human-disturbed habitats and agricultural land [43] and naturally occurring dry habitats [35]. The species is native throughout Europe, and it has naturalized in temperate Asia [44]. Research on pyrrolizidine alkaloids (PAs) in *M. arvensis* remains scarce, underscoring the relevance of this study. As members of the *Boraginaceae* family, *Myosotis* species are recognized PA producers [45,46]. While PA occurrence within the genus is documented [47,48], species-specific data and concentration levels are limited. Earlier studies in the United States on *M. scorpiodes* and *M. sylvatica* identified both monoester and diester PAs, including heliosupine and its N-oxide, indicating a conserved biosynthetic pathway within the genus [48,49].

Considering the ecological significance, toxicological impact, and environmental persistence of pyrrolizidine alkaloids described above, the aim of this study was to investigate the leaching behavior of toxic pyrrolizidine alkaloids—regulated by Commission Regulation (EU) 2023/915 [12]—in two different soil types over time, using plant extracts from the model species *Myosotis arvensis* L. and *Senecio vulgaris* L., which represent two major PA-producing families—Boraginaceae and Asteraceae—prepared through hot and cold extraction methods.

## 2. Results

### 2.1. PA Content in Weed Extracts

**Figure 1:**
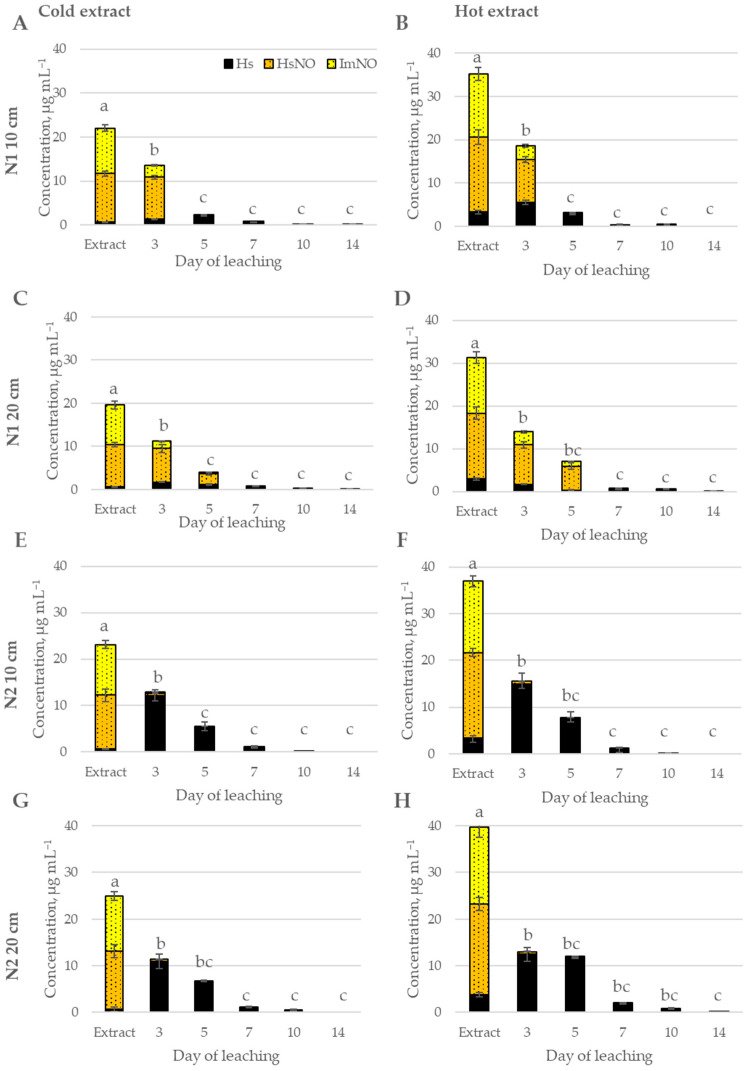
Leaching of intermedine-N-oxide (ImNO), heliosupine N-oxide (HsNO), and heliosupine (Hs) from *Myosotis arvensis* extracts in soil. (**A**)—soil N1 10 cm, cold extract; (**B**)—soil N 10 cm, hot extract; (**C**)—soil N1 20 cm, cold extract; (**D**)—soil N1 20 cm, hot extract; (**E**)—soil N2 10 cm, cold extract; (**F**)—soil N2 10 cm, hot extract; (**G**)—soil N2 20 cm, cold extract; (**H**)—soil N2 20 cm, hot extract. Means marked with the same letter within each graph are not significantly different at *p* ≤ 0.05 according to Duncan’s multiple range test.

**Figure 2:**
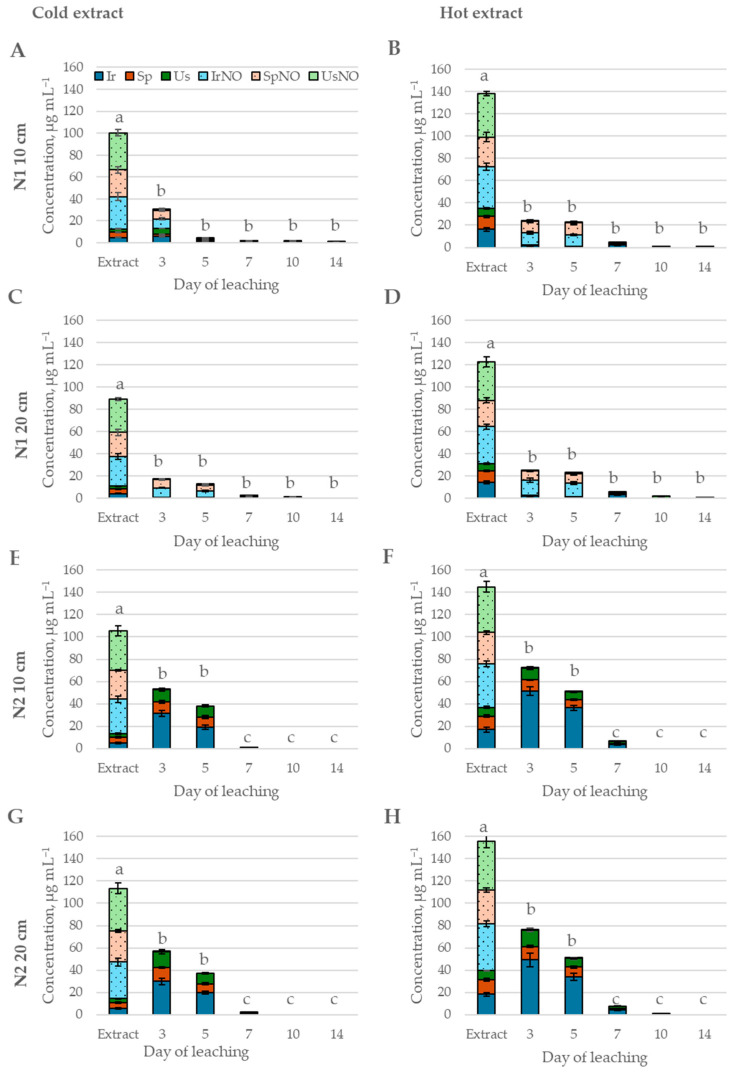
Leaching of Ir, Sp, Us, IrNO, SpNO, and UsNO from *Senecio vulgaris* extracts in soil. (**A**)—soil N1 10 cm, cold extract; (**B**)—soil N 10 cm, hot extract; (**C**)—soil N1 20 cm, cold extract; (**D**)—soil N1 20 cm, hot extract; (**E**)—soil N2 10 cm, cold extract; (**F**)—soil N2 10 cm, hot extract; (**G**)—soil N2 20 cm, cold extract; (**H**)—soil N2 20 cm, hot extract. Means marked with the same letter within each graph are not significantly different at *p* ≤ 0.05 according to Duncan’s multiple range test.

Two pyrrolizidine alkaloid-containing weed species, *Myosotis arvensis* (MA) and *Senecio vulgaris* (SV), which are widespread in agricultural fields, were used as model plants representing the most common PA-producing families—Boraginaceae and Asteraceae—in a soil fate experiment focused on PAs regulated by Commission Regulation (EU) 2023/915. In MA water extracts, three PAs were detected, intermedine-N-oxide (ImNO), heliosupine N-oxide (HsNO), and heliosupine (Hs) (Figure 1), while in *S. vulgaris* (SV) water extracts, six were detected: seneciphylline (Sp) and its N-oxide (SpNO), integerrimine (Ir) and its N-oxide (IrNO), and usaramine (Us) and its N-oxide (UsNO) (Figure 2). The total PA concentration significantly differed between hot and cold extractions (*p* < 0.001), with extraction at higher temperatures increasing total PA content by 60% in MA and by 37% in SV (Supplementary Table S1). As expected, higher PA concentrations were observed in hot extracts, as temperature is known to influence phytochemical extraction efficiency, composition, and solubility [50]. In this case, hot extraction enhanced PA solubility and increased yield by promoting the breakdown of plant cell walls, thereby facilitating their release.

### 2.2. Fate of Pyrrolizidine Alkaloids in Soil

At the beginning of the experiment, the presence of PAs was analyzed in both the soil used and the water used to prepare the extracts. The results confirmed that neither the soil nor the water contained detectable levels of PAs. Among all experiments involving the extraction of PA-containing weed extracts, additional tests were conducted by rinsing the soil with water. PAs were not present in any of the soil types studied.

#### 2.2.1. *Myosotis arvensis* Cold and Hot Extracts

**Figure 3:**
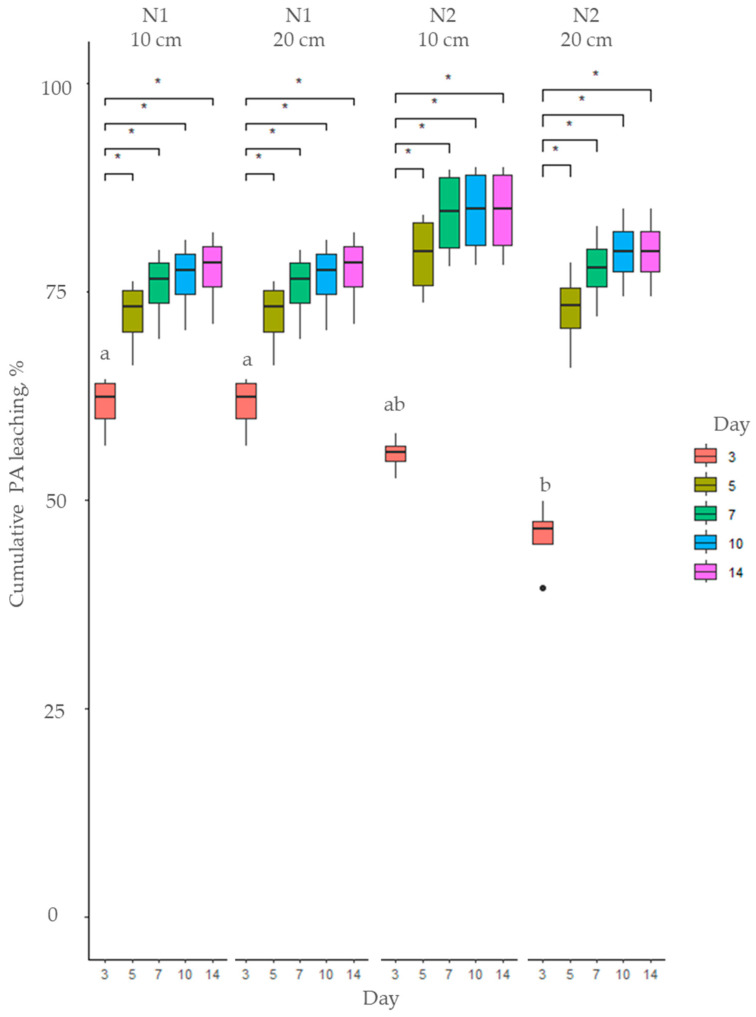
Cumulative PA leaching (%) of total PAs from soil using *Myosotis arvensis* cold extracts: soil N1—sandy loam; soil N2—loam. Means marked with the same letter are not significantly different at *p* ≤ 0.05 according to Duncan’s multiple range test. The asterisks indicate statistically significant differences (* *p* < 0.05).

**Figure 4:**
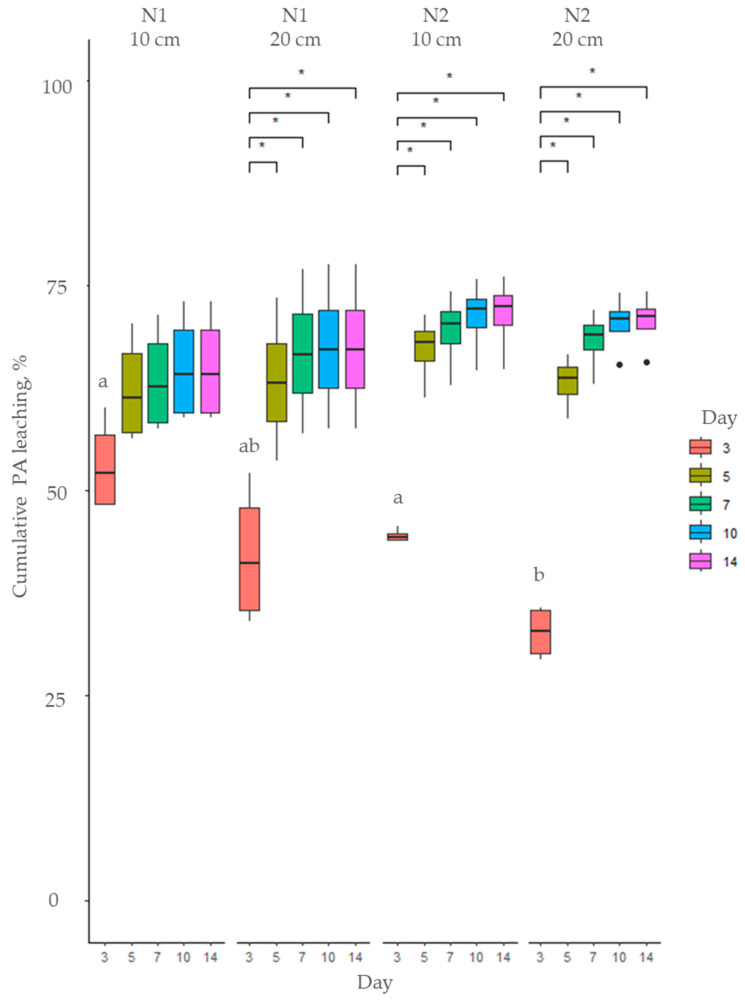
Cumulative PA leaching (%) of total PAs from soil using *Myosotis arvensis* hot extracts: soil N1—sandy loam; soil N2—loam. Means marked with the same letter within each graph are not significantly different at *p* ≤ 0.05 according to Duncan’s multiple range test. The asterisks indicate statistically significant differences (* *p* < 0.05).

Hot and cold extracts prepared from *M. arvensis* (MA) were applied to soil and over a 14-day period, the leaching of total PAs was recorded, as illustrated in Figure 3 and Figure 4. The cumulative proportion of leached PAs was significantly higher when cold extracts were applied to soil (*p* < 0.001) compared to hot extracts throughout the entire 14-day period. However the hot extracts had a higher total PA concentration (Figure 1), and a smaller proportion of PAs applied to soil leached from day 3 to 14 (Figure 3 and Figure 4, Supplementary Table S1). The leaching of both hot and cold MA extracts was influenced by soil type and soil layer only on day 3 (*p* < 0.001). However, in the subsequent days, the cumulative leaching of total PAs was not influenced by soil type or soil layer (Figure 3 and Figure 4, Supplementary Table S1).

After application of MA cold extracts, PA leaching from the soil was most prominent during the first three days, followed by a steady leaching phase until day 5, after which it stabilized, reaching a plateau (Figure 3). By day 14, 77–84% of the initial amount of PAs in the extract had leached from the soil.

Similarly, the leaching of MA hot extracts was rapid initially, up to day 5, and stabilized afterwards (Figure 4). Over the first five days, the cumulative leaching accounted for 62–67% of the PA content in the applied extract across both soil types and depths. By day seven, the leaching rate slowed, with total cumulative leaching ranging from 63% to 69%, showing only a minimal increase in the subsequent days, reaching 65—71% by day 14.

**Figure 5:**
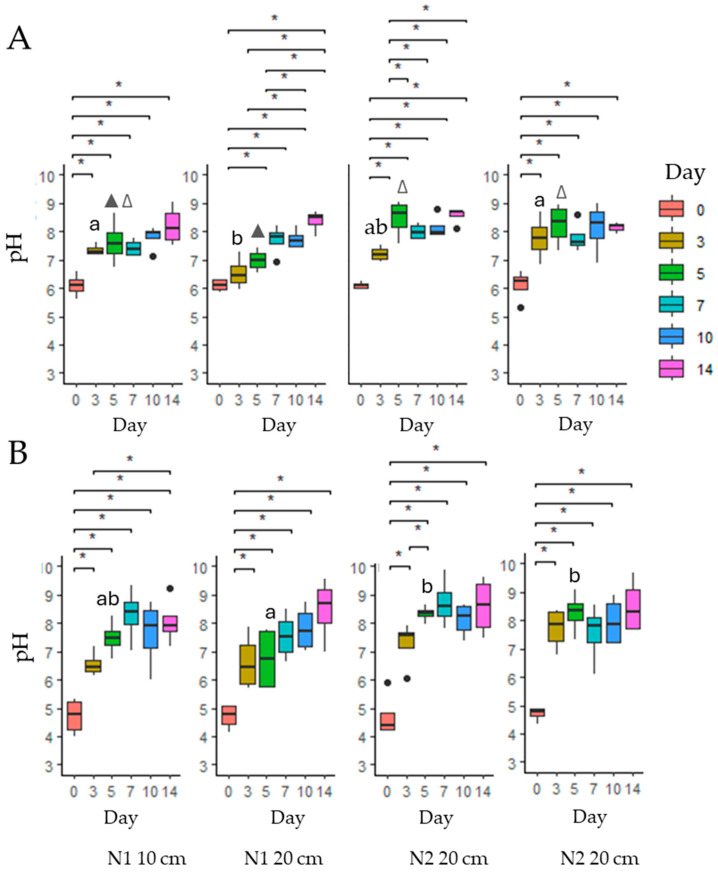
pH values of *Myosotis arvensis* PA extracts and corresponding leachates over a 14-day period. (**A**)—cold MA extracts; (**B**)—hot MA extracts. Means marked with the same letter or same symbol (∆▲) within each row of graphs (**A**,**B**) are not significantly different at *p* ≤ 0.05 according to Duncan’s multiple range test. The asterisks indicate statistically significant differences (* *p* < 0.05).

The individual PAs of the *M. arvensis* extracts—heliosupine (Hs), heliosupine N-oxide (HsNO), and intermedine N-oxide (ImNO)—exhibited distinct leaching patterns over the 14-day period across different soil types, depths, and weed extraction conditions (Figure 5).

Both PANOs (HsNO and ImNO) accounted for the majority of the PA content in extracts applied to the soil at the start of the experiment, representing 97% in CE and 91% in HE (Figure 1). Three days after the extract was applied to the soil, the leachate contained a lower concentration of PANOs and a higher concentration of free-base PAs than the applied extract. This indicates that in the soil, under anaerobic and reducing conditions, the PANO (HsNO) was converted into the more stable free-base PA—Hs. This conversion was more pronounced in loam soil (N2) compared to sandy loam soil (N1). The leachates did not contain any ImNO by day 5 in N2 soil and by day 7 in N1 soil. Similarly, the leachates did not contain any HsNO by day 10. The free-base form Hs persisted in the leachates longer.

After 14 days, no PAs were detected in leachates of soil N2, while residual PAs persisted in soil N1, indicating that PA degradation is influenced by soil type. Despite near-zero PA concentrations in leachates, residual PAs were still detectable in soil samples, with no N-oxides found in either leachates or soil.

According to Figure 5, the MA hot extract had a significantly lower pH than the cold extract, classifying it as acidic (*p* < 0.001) (Supplementary Table S2). Over the 14-day period, the pH of the leachates changed to alkaline, and by day 7, no significant differences among extraction type, soil type, or soil layer were observed. However, soil type influenced pH changes in the leachate measured on day 3 and day 5, as the leachate from N2 soil had a significantly higher pH than the leachate from N1 soil (*p* < 0.01) (Supplementary Table S2).

After 14 days of leaching with *Myosotis arvensis* extracts, heliosupine (He) was detected in the soil, whereas none of the PANOs present in the extracts were found. The hot extract (HE), which contained a higher concentration of PAs, also resulted in a higher residual concentration of PAs in the soil (Table 1).

#### 2.2.2. *Senecio vulgaris* Cold and Hot Extracts

**Figure 6:**
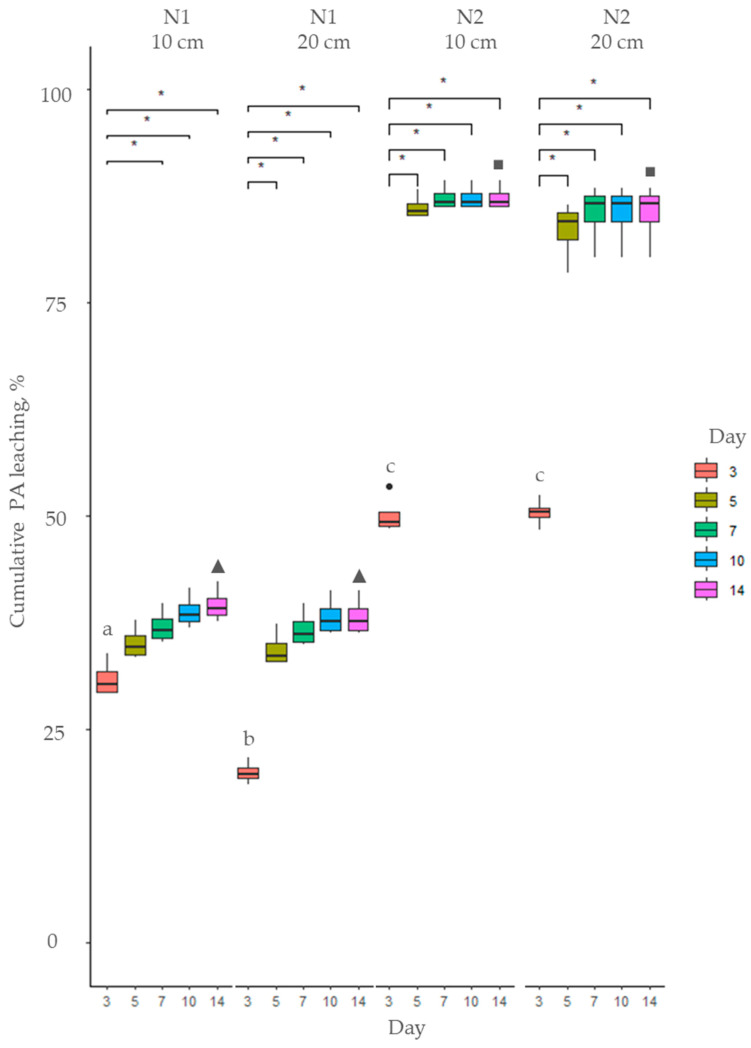
Cumulative PA leaching (%) of total PAs from soil using *Senecio vulgaris* cold extracts: soil N1—sandy loam; soil N2—loam. Means marked with the same letter or same symbol (▲■) are not significantly different at *p* ≤ 0.05 according to Duncan’s multiple range test. The asterisks indicate statistically significant differences (* *p* < 0.05).

**Figure 7:**
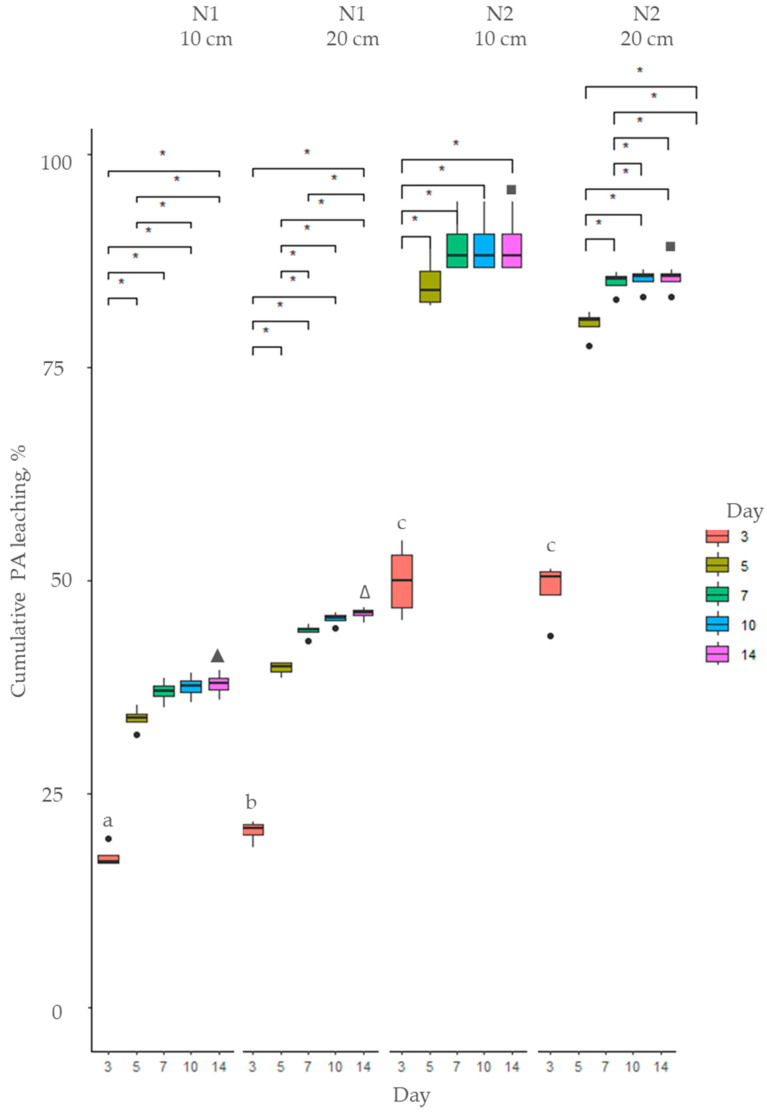
Cumulative PA leaching (%) of total PAs from soil using *Senecio vulgaris* hot extracts: soil N1—sandy loam; soil N2—loam. Means marked with the same letter or the same symbol (∆▲■) not significantly different at *p* ≤ 0.05 according to Duncan’s multiple range test. The asterisks indicate statistically significant differences (* *p* < 0.05).

The PAs and PANOs leached from the soil steadily within the first 5 days after application of CE and HE of *S. vulgaris* and stabilized afterwards (Figure 6 and Figure 7). After 14 days, no PAs or PANOs were detected in leachates from N2 soil, and minor leaching was still present from N1 soil (<0.84% from the concentration of PAs and PANOs in the extract). Both the extraction type (*p* < 0.05) and soil (*p* < 0.001) had significant effect on the amount of PAs and PANOs leaching throughout the 14-day period (Supplementary Table S3).

The hot extract initially had a 27% higher content of PAs and PANOs (Figure 2); thus, the leached concentrations of the total amount of PAs and PANOs were higher when the HE was applied. To account for the initial differences in the concentration of solutes in both extract types, the percentage of PAs and PANOS in the leachate was calculated relative to the initial concentration in the extract. On average, a significantly (*p* < 0.05) higher proportion of solutes was leached from the CE up to day 5, but afterwards, higher leaching was observed from the HE (Supplementary Table S3).

**Figure 8:**
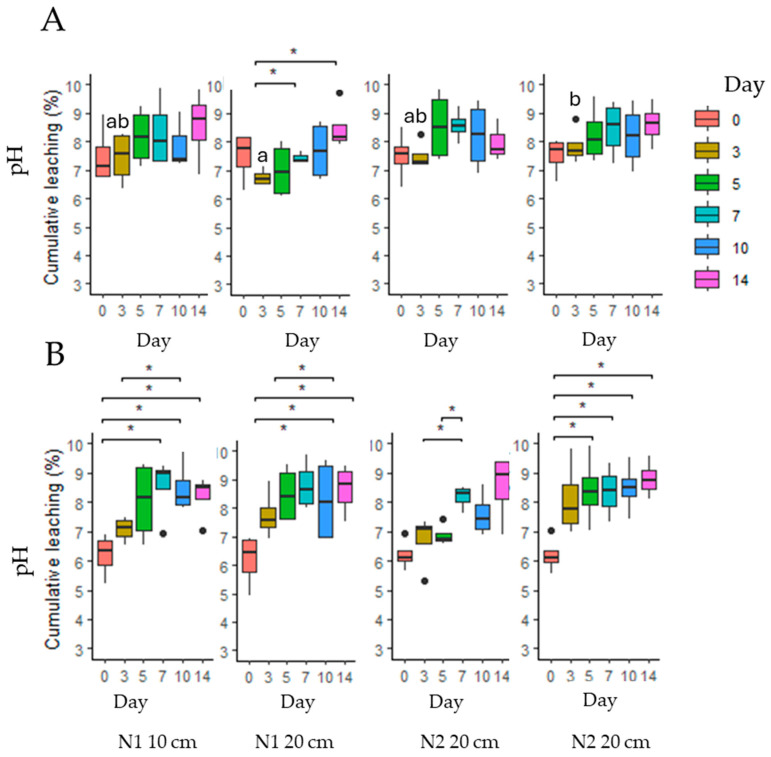
pH values of *Senecio vulgaris* PA extracts and corresponding leachates over a 14-day period. (**A**)—cold MA extracts; (**B**)—hot MA extracts. Means marked with the same letter within each row of graphs (**A**,**B**) are not significantly different at *p* ≤ 0.05 according to Duncan’s multiple range test. The asterisks indicate statistically significant differences (* *p* < 0.05).

The PA content in the SV extracts was mainly PANOs (IrNO, SpNO, and UsNO), accounting for 87% in CE and 74% in HE (Figure 2), while the rest consisted of free-base PAs. During the first three days after the extract was applied to the soil, the PANO concentration significantly decreased (Figure 7). The concentration of IrNO and UsNO fell below the detection threshold by day 3 in leachates from N2 soil; however, they persisted in leachates from N1 soil up to day 10. SpNO remained detectable in leachates from both soils up to day 10. Meanwhile, changes in the concentration of free-base PAs differed between N1 and N2 soil. In N1 soil, the amount of free-base PAs decreased, whereas in N2 soil, the concentration of each PANO was higher in the leachate on day 3 compared to the applied extract. In the following days, PAs leached more rapidly from the N2 soil, falling below the detection limit by day 10, while residual amounts remained in leachates from N1 soil through day 14 (Figure 8).

The pH of the SV hot extract was significantly (*p* < 0.001) lower than that of the cold extract, making it acidic (Figure 8). Over the 14-day period, the pH of the leachates shifted to alkaline, and by day 7, no notable differences were found between extraction type, soil type, or soil layer (*p* > 0.05). However, soil type did affect pH changes in the leachate of cold extract measured on day 3, with the leachate from N2 soil showing a significantly higher pH compared to that from N1 soil (*p* < 0.05) (Supplementary Table S4).

The soil after 14 days of leaching *Senecio vulgaris* extracts contained all PAs that were in the extract—Ir, Sp, and Us. Their respective PANOs—IrNO and UsNO—were not detected in the soil, but a small residual amount of SpNO was detected in the soil leached with an extract of higher PA concentration (HE) (Table 2).

## 3. Discussion

The aim of this study was to understand the behavior and movement of toxic pyrrolizidine alkaloids as they enter soil systems from different PA-containing weed species, focusing exclusively on the PAs regulated by Commission Regulation (EU) 2023/915 [12]. Two dominant soil types found in Latvia (sandy loam and loam) and two of the most common PA-producing weeds in Latvian agriculture—*M. arvensis* (MA) and *S. vulgaris* (SV), representing the major PA-containing plant families Boraginaceae and Asteraceae—were purposefully selected.

### 3.1. Pyrrolizidine Alkaloid Profiles in Myosotis arvensis and Senecio vulgaris

Analysis of pyrrolizidine alkaloids (PAs) monitored under Commission Regulation (EU) 2023/915 revealed that *Myosotis arvensis* grown in Latvia contained intermedine-N-oxide (ImNO), heliosupine N-oxide (HsNO), and heliosupine (Hs). The available literature on PAs in *M. arvensis* is rather limited; therefore, this study provides novel data on the occurrence and concentration of selected PAs in this species. This species belongs to the Boraginaceae family, which is known for synthesizing PAs across all its genera [45,46]. Sources compiling information on PA occurrence in various plants confirm the presence of these alkaloids within the Myosotis genus [47,48]; however, data on specific species and their PA concentrations remain scarce. Studies on *M. scorpioides* and *M. sylvatica*, conducted in the United States about forty years ago, indicate that species within this genus can contain both PA monoesters and diesters [48,49]. This finding aligns with our results, as both esterification forms of PAs were detected in the samples analyzed. Among the identified compounds, Hs and its N-oxide HsNO were also found in our MA extracts, suggesting a possible shared genetic factor within the *Myosotis* genus.

The *Senecio* genus is well-documented in the scientific literature regarding PAs, covering aspects ranging from quantitative PA content [38,39] to biosynthesis pathways [4]. This extensive research interest is likely influenced by historical factors, particularly the fact that *Senecio* species contain significantly higher PA concentrations compared to other PA-producing plants. Additionally, these species synthesize some of the most toxic PA types, which align with the data obtained in our study. Six PAs were detected in *S. vulgaris* grown in Latvia: seneciphylline (Sp) and its N-oxide (SpNO), integerrimine (Ir) and its N-oxide (IrNO), and usaramine (Us) and its N-oxide (UsNO). Moreover, *Senecio* plants are widely distributed across almost all regions of the world, further contributing to their scientific relevance. Notably, SpNO, IrNO, and UsNO have been detected in various plant parts. For instance, a study analyzing PA variation in *S. vulgaris* populations found that SpNO and IrNO were dominant in the roots, while UsNO was detected less frequently [38,39].

### 3.2. Soil Type Effects on Pyrrolizidine Alkaloid Leaching Dynamics

It is well established that the leaching of PAs from soil is influenced by precipitation levels [25,26]. In Latvia, the average annual precipitation is approximately 685.6 mm, with seasonal averages of about 123.1 mm in spring, approximately 222.6 mm in summer, and around 195.3 mm in autumn. According to the Latvian Environment, Geology and Meteorology Centre, the highest monthly rainfall typically occurs in July and August, with averages of 75.7 mm and 76.8 mm, respectively [51]. Our study was based on continuous soil leaching, with soil moisture saturation maintained at approximately 85% over a 14-day period. This corresponded to a precipitation depth ranging from 79.6 to 162.7 mm, depending on the soil type and soil layers (10 cm and 20 cm).

The leaching dynamics of total PAs were strongly influenced by both the plant species and the extraction method. Extracts from *M. arvensis* exhibited a clear difference between cold and hot preparations: cold extracts resulted in significantly higher cumulative leaching (77–84%) compared to hot extracts (65–71%) after 14 days. This effect is attributed to the greater proportion of water-soluble N-oxides (e.g., HsNO and ImNO) present in cold extracts, which leached more readily due to their higher polarity [52]. In contrast, hot extracts were enriched in open-chain diester free-base PAs (e.g., Hs), which interact more strongly with soil components and thus exhibit lower mobility. These results indicate that elevated temperatures enhance plant cell wall disruption and increase the solubility of free-base PAs, improving extraction efficiency [53]. In the case of *M. arvensis*, soil type affected PA mobility only during the first three days of leaching, with sandy loam (N1) showing slightly higher leaching than loam (N2); however, by day 14, these differences were no longer significant.

In contrast, leaching from *S. vulgaris* extracts was markedly lower under all conditions. After 14 days, cumulative leaching was below 0.84% in sandy loam (N1), and no PAs or PANOs were detected in leachates from loam soil (N2). This pattern was consistent for both cold and hot extracts. Although the hot extract of SV had 27% higher initial PA and PANO content, cumulative leaching was not proportionally greater. When normalized to initial extract concentrations, significantly more solutes leached from cold extracts during the first five days (*p* < 0.05), while hot extracts contributed marginally more to cumulative leaching afterward. The substantially lower mobility of PA from SV extracts is likely due to their chemical structure: all PAs detected (e.g., Sp, Ir, and Us) are cyclic diesters, which form stronger interactions with soil particles and degrade more rapidly under loam conditions. This is further supported by the presence of residual compounds in sandy loam (N1) and their complete degradation in loam (N2) by day 14. Therefore, both the chemical structure (cyclic vs. open-chain diesters) and soil type (N1 vs. N2) played decisive roles in determining PA behavior in soil systems for both studied weed species.

### 3.3. Influence of Soil Physicochemical Properties

#### 3.3.1. pH, Organic Matter, and Soil Texture Effects

The fate of PAs in soil can be influenced by various factors. It has been reported that soil pH affects the ionization state of PAs and PANOs, thereby altering their overall mobility [20,54]. Consequently, it cannot be excluded that pH may also enhance adsorption to soil particles. Based on the findings of this study, three main factors can be identified regarding the influence of pH: the plant extracts themselves, the soil properties, and the water used for leaching (or, under natural conditions, rainfall). Typically, rainfall has a pH of around 5.6; however, this can decrease further and become more acidic when significant amounts of atmospheric pollutants such as CO_2_, NO, and SO_2_ react with water in the atmosphere [55]. In our study, the water used for both extract preparation and the leaching process had a pH close to that of natural rainwater (approximately 5.5). As this parameter remained constant throughout the entire experiment, its influence on the results can be considered negligible. Notable pH patterns were observed among the PA-containing weed extracts. Across all four prepared extracts, a consistent trend emerged: hot-water extracts exhibited lower pH values compared to their cold counterparts. Specifically, *M. arvensis* hot extract showed a pH of 4.1, and *S. vulgaris* hot extract had a pH of 6.2, whereas the corresponding cold extracts had pH values of 6.1 and 7.5, respectively. The markedly low pH of *M. arvensis* hot extract is noteworthy, as it approaches the acidic range. This temperature-dependent pH shift can be attributed to the thermal hydrolysis of plant cell wall components, particularly hemicellulose. Hemicellulose is a heteropolymer composed of various sugar units such as xylans, mannans, and glucans, which form an integral part of the plant cell wall structure [56]. Under elevated temperatures, portions of hemicellulose undergo hydrolysis, releasing organic acids such as acetic acid. These acids contribute to a decrease in pH and further catalyze hemicellulose degradation [57]. Such acidification during hot extraction not only reflects chemical changes within the plant matrix but may also influence the stability and environmental behavior of the plant’s chemical compounds—including pyrrolizidine alkaloids—especially during leaching processes. However, once these extracts entered the soil and interacted with it, the pH values of the leachates obtained on day 3 increased rapidly, ranging from 6.5 to 8.1. The average pH values continued to rise gradually, reaching up to 8.5 by day 14—ranging from 8.4 in sandy loam soil (N1) to 8.6 in loam soil (N2).

The mobility and uptake of PAs in plants is significantly influenced by soil pH, which alters the solubility and availability of these compounds [58]. In acidic environments, PAs generally exist in a protonated form, while under alkaline conditions, they remain non-protonated. These different ionization states affect how easily PAs can penetrate plant membranes, thereby influencing their absorption and translocation within plant tissues [54]. However, uptake by plants is not a straightforward process; rather, it is governed by sorption and desorption mechanisms [22,23]. Initially, PAs released from weeds bind to soil particles. Through desorption, they may subsequently become available for uptake by other plants or be transported further into groundwater via precipitation and leaching.

#### 3.3.2. Sorption, Desorption, and Mobility Mechanisms

Recent studies have placed particular emphasis on the behavior of PAs in soil environments [22,23,59]. These investigations show that PAs and their N-oxides (PANOs) exhibit weak sorption and high desorption, especially under acidic conditions and in soils with low organic matter. Sorption is both pH-dependent and reversible, allowing these compounds to remain mobile in the environment [59]. PANOs, due to their higher polarity, demonstrate even lower sorption affinity than their parent PAs, resulting in greater leaching potential and bioavailability [22]. Desorption experiments further confirm that a significant fraction of previously sorbed PAs and PANOs can be remobilized, particularly in acidic soils [23]. Additionally, Kisielius and colleagues reported that PAs can leach into soil and water systems, with their persistence and movement affected by both soil composition and broader environmental factors [16]. These findings underscore that soil properties—such as pH, organic carbon content, and cation exchange capacity (CEC)—are key determinants of PA fate, and the reversibility of sorption amplifies environmental risks by facilitating plant uptake and groundwater contamination. In our study, although the two soils had different pH values—6.0 (in H_2_O) for the sandy loam (N1) and 8.3 for the loam (N2)—neither could be classified as acidic; however, the loam trended toward the alkaline range. The loam soil also contained substantially higher concentrations of magnesium and calcium salts, resulting in a CEC approximately four times greater than that of the sandy loam. Regarding textural composition, both soils had the same clay content, but differed in their sand and silt fractions. In the loam soil (N2), sand and silt were nearly equal (48% and 43%, respectively), whereas the sandy loam (N1) contained 73% sand and 18% silt. As mentioned earlier, by day 14, 77–84% of the total amount of PAs from *M. arvensis* extracts had leached from the soil with cold extracts, while 65–75% had leached using hot extracts. These findings demonstrate that soil composition and depth significantly influence PA leaching rates during the initial days, when leaching is most pronounced. However, this effect diminishes over time, with minimal impact on PA leaching in the long term, even at depths of up to 20 cm. In contrast, *S. vulgaris* exhibits distinct leaching patterns, indicating a stronger dependency on soil properties and potentially different interactions with soil components. Soil type (*p* < 0.001) had a significant effect on the amount of PAs and PANOs leaching throughout the 14-day period, and clear differences between soils were observed. By day 14, no PAs or PANOs were detected in the leachates from loam soil (N2), while in sandy loam soil (N1), cumulative leaching remained minimal, below 0.84% of the total PA and PANO content applied. This indicates that *S. vulgaris*-derived PAs show very limited mobility in both soil types, with faster degradation or stronger retention, especially in loam soil.

The results of this study revealed that PAs, particularly those derived from *S. vulgaris*, leached faster from loam soil (N2) compared to sandy loam (N1). This observation is intriguing given that loam soil (N2) exhibited a lower organic matter content (1.9%) compared to sandy loam (2.5%). Typically, organic matter is known to enhance the sorption of phytotoxic compounds through strong hydrogen bonding and hydrophobic interactions, contributing to reduced mobility in soils [60]. However, the faster leaching observed in loam (N2) can be attributed to its significantly higher cation exchange capacity (CEC) of 19.7 cmol (+)/kg compared to 4.7 cmol (+)/kg in sandy loam (N1). Higher CEC values indicate a greater density of negatively charged sites on soil particles, which can bind to positively charged ions and organic molecules, including PAs. In the case of *S*. *vulgaris*, which predominantly produces cyclic diester PAs, the increased electrostatic interactions facilitated by the elevated CEC in N2 may enhance temporary sorption but do not prevent overall mobility. This may suggest that while initial retention is strong, subsequent displacement through competitive ion exchange or solubilization in the soil solution accelerates leaching, particularly in the upper soil layers.

Moreover, loam soil (N2) contained substantially higher levels of calcium (2259 mg·kg^−1^) compared to sandy loam (783 mg·kg^−1^). Calcium ions (Ca^2+^) are known to influence the stability of soil aggregates and promote stronger sorption through the bridging of organic molecules and soil particles [35]. Despite the expectation of stronger PA retention, the observed higher leaching rates from N2 suggest that calcium-driven aggregation may enhance early-phase displacement of PAs, particularly under wet conditions where ionic competition is prevalent. In contrast, sandy loam (N1), with its higher sand content (73%), exhibited different sorption behavior. While sand generally increases soil permeability and decreases water retention, PAs derived from *S*. *vulgaris* were observed to be retained longer in N1. This aligns with findings from Mrkajic and team, who noted that biological sand filters are effective for the removal of phytotoxins in drinking water but are less effective at eliminating pyrrolizidine alkaloids, as these compounds tend to persist in the filter media [28]. This persistence suggests that while sand-dominant soils may not strongly sorb PAs, their physical structure may still trap and slow down the migration of these alkaloids compared to loam, where higher ionic competition facilitates quicker release. The combined effects of high CEC and elevated calcium levels in loam soil may create dynamic interactions where PAs are initially adsorbed but remain vulnerable to mobilization upon changes in soil moisture or ionic competition. These findings indicate that while loam soil possesses a strong initial sorption capacity, its physicochemical properties may paradoxically facilitate greater PA mobility, especially for cyclic diester PAs from *S. vulgaris*. This highlights the complexity of PA–soil interactions and underscores the need for further studies focusing on competitive sorption and ionic displacement mechanisms in varying soil types.

### 3.4. Reduction of PANOs and Toxicological Impact of PA Chemical Structures

It is well established that pyrrolizidine alkaloids (PAs) in plants mostly exist in two forms: the parent PA and its N-oxide (PANO) [61]. N-oxidation is a reversible modification that significantly alters the physicochemical properties of the native PAs, making PANOs more polar and highly water-soluble. In plant tissues, the majority of PAs are present in the N-oxide form [4,62]. However, due to the reversibility of N-oxidation, PANOs can be converted back to their less water-soluble parent PA forms under various conditions, and in multiple environments, including during ensiling [63], composting [64], and in vitro rumen fermentation [61], and within the rat intestine [65].

This phenomenon was also observed in our study, where a portion of the PANO compounds present in the extracts transformed into their corresponding parent PAs upon entering the soil. The leaching experiments with *M. arvensis* and *S. vulgaris* extracts clearly demonstrated this conversion, which was particularly evident in both soil types (N1—sandy loam; N2—loam). The initial concentration of N-oxides (e.g., HsNO and ImNO for *M. arvensis* and SpNO, IrNO, and UsNO for *S. vulgaris*) was substantially higher in the extracts compared to the parent PAs (Hs, Sp, Ir, and Us). However, during the first three days of leaching, a sharp decline in N-oxides was accompanied by a relative increase in the concentration of their corresponding parent PAs in the leachates.

This shift was more pronounced in *S. vulgaris* extracts, where SpNO, IrNO, and UsNO exhibited rapid reduction, with a detectable increase in their parent forms (Sp, Ir, and Us), especially in loam soil (N2). These findings suggest that the soil environment promoted reductive conditions that favored the transformation of N-oxides back to free-base PAs, a process potentially driven by microbial activity or soil-mediated redox reactions. Notably, this transformation was faster and more complete in the loam soil (N2), characterized by a higher CEC and calcium content, which may facilitate microbial activity and redox cycling. In contrast, sandy loam (N1) exhibited slower conversion rates, possibly due to lower organic matter and reduced microbial biomass. These observations are consistent with previous studies indicating that soil composition and physicochemical properties significantly influence the stability and mobility of PAs and their N-oxides [61,63,64]. Earlier studies also report that ImNO and HsNO degrade rapidly under aerobic conditions, first converting to their parent PAs before undergoing further breakdown. Heliosupine degrades more slowly but ultimately transforms into less toxic by-products within 14–21 days [66]. These results align with our findings, showing accelerated PA degradation in loam soil compared to sandy loam, highlighting the role of soil composition in PA transformation. Additionally, the chemical structure of PA compounds plays a critical role in their environmental behavior. In *M. arvensis*, the identified PAs are either monoesters or open-chain diesters, where N-oxides contain multiple labile bonds that, when cleaved, can lead to the formation of stable PA structures. Conversely, in *S. vulgaris*, the PAs are cyclic diesters, which lack such reactive sites, making the transformation of N-oxides into stable PA forms significantly more challenging. This structural rigidity likely contributes to the lower conversion rates observed in *Senecio*-derived PANO compounds during leaching.

### 3.5. Environmental Risk and Future Directions

The toxicity and environmental behavior of PAs in this study were closely linked to their chemical structures and interactions with soil components. As shown in Table 3, which presents information on the esterification types of the individual PAs—a key factor in assessing their toxicity [9]—*Senecio vulgaris* extracts were dominated by cyclic diester (CDE) PAs such as Sp, Ir, and Us. These compounds are classified as highly toxic [2,9] and are considered the most hazardous PA class based on relative potency (REP) values [17]. Despite their high toxicity, these compounds exhibited limited mobility, particularly in loam soil (N2), where no PAs or PANOs were detected in leachates by day 14. In contrast, *Myosotis arvensis* extracts contained open-chain diesters (DEs) such as Hs and its N-oxide (HsNO), as well as monoesters (MEs) like ImNO. These are considered less toxic [2,9] but showed significantly higher leaching, especially in sandy loam soil (N1). Notably, PANOs—initially more polar and less toxic—underwent partial conversion to their corresponding parent PAs in both soil types, with the transformation more pronounced in loam (N2), suggesting redox activity in the soil environment [20]. This is environmentally concerning, as PANOs can revert to more bioavailable and toxic forms under favorable conditions. While loam soil tended to promote both PA retention and transformation, sandy loam tended to retain residual compounds—particularly Hs—for a longer duration. These findings emphasize the need for soil-specific risk assessments, given that both structure-dependent toxicity and dynamic transformations influence the environmental fate of PAs.

## 4. Conclusions

This study provides critical insights into the environmental fate of toxic pyrrolizidine alkaloids regulated under Commission Regulation (EU) 2023/915 by examining their leaching behavior from two representative PA-producing species—*Myosotis arvensis* and *Senecio vulgaris*—in two distinct soil types. The results demonstrate that both the chemical structure of PAs and soil properties significantly influence their mobility, transformation, and persistence. Leaching of PAs from *M. arvensis* cold-water extracts, dominated by highly polar N-oxides, was significantly faster (77–84% within five days) compared with hot-water extracts (62–67%). In contrast, cyclic diesters from *S. vulgaris* exhibited substantially lower mobility (<0.84% cumulative leaching), attributed to strong sorption and rapid degradation, with no detectable residues in loam soil by day 14 and only minor persistence in sandy loam soil. Loam soil, characterized by a higher cation exchange capacity and calcium content, strongly promoted both the reduction of PANOs (e.g., over 90% conversion of heliosupine N-oxide to heliosupine within five days) and PA sorption, whereas sandy loam soil facilitated greater leaching and prolonged retention, with residues detectable even after 14 days. The reductive transformation of N-oxides to more toxic free-base PAs underscores the need for soil-type-specific monitoring of both parent PAs and their N-oxides. Given the known hepatotoxicity, genotoxicity, and carcinogenic potential of these compounds, especially cyclic diesters, their demonstrated persistence and potential to reach groundwater sources raise significant environmental and public health concerns. Future research should address PA transport beyond topsoil and clarify the microbial and physicochemical processes governing their fate in deeper soils and aquatic systems. This study represents an important next step toward advancing the understanding and environmental risk assessment of PA contamination.

## 5. Materials and Methods

### 5.1. Reference Standards and Reagents

Deionized water (18.2 MΩ*cm) was obtained from Thermo Scientific Barnstead Smart2Pure 6 UV (Waltham, MA, USA). Acetonitrile (CH_3_CN), formic acid (HCOOH), methanol (CH_3_OH), and ammonium formate (HCOONH_4_) were purchased from Fisher Scientific (Waltham, MA, USA). Magnesium sulfate (MgSO_4_, anhydrous, 99.5%) was obtained from Alfa Aesar (Haverhill, MA, USA). Sodium chloride (NaCl, 99.5%) and disodium hydrogen citrate sesquihydrate (Na_2_C_6_H_6_O_7_) were purchased from Acros Organics (Geel, Belgium). Trisodium citrate dihydrate (C_6_H_5_Na_3_O_7_∙2H_2_O ≥ 99.5%) was obtained from Fisher Scientific (Waltham, MA, USA). The reference solution for mass spectrometry, containing purine (*m*/*z*—121.0509) and hexakis (*m*/*z*—922.0098), was purchased from Agilent Technologies (Santa Clara, CA, USA). Intermedine-N-oxide (ImNO) (96.79%), integerrimine (Ir) (97.67%), integerrimine-N-oxide (IrNO) (96.63%), seneciphylline (Sp) (99.13%), seneciphylline-N-oxide (SpNO) (99.17%), usuramine (Us) (97.68%), usuramine-N-oxide (UsNO) (97.52%), Hheliosupine sulfate (Hs) (99.10%), and heliosupine-N-oxide (HsNO) (96.40%) were purchased from PhytoLab GmbH & Co. KG (Vestenbergsgreuth, Germany).

### 5.2. Preparation of Pyrrolizidine Alkaloid-Containing Plant Extracts

#### 5.2.1. Weed Samples

*M. arvensis* (MA) was collected on 16 June 2023 from an agricultural field (57°12′10.7″ N 25°08′41.9″ E), and *S. vulgaris* (SV) was collected on 5 July 2023 from a garden (57°18′49.4″ N 25°18′21.8″ E). After collecting, the weed samples were cleaned of sand, cut into smaller pieces using garden shears, and then dried for 48 h at 55 °C in a drying oven. After drying, the samples were stored in sealed, high-density, dark polyethylene containers until analysis. Prior to analysis, the samples were ground and homogenized.

#### 5.2.2. Cold (CE) and Hot (HE) Extracts

The use of hot and cold weed extracts in PA leaching experiments is important because temperature affects the extraction efficiency, composition, and solubility of PAs, which can influence their environmental behavior. The cold extraction process was performed using 201.71 g of MA and 125.93 g of SV in 4000 mL of distilled water, followed by maceration in a dark, cool place for 7 days. After 7 days, the extracts were mixed and filtered. For hot extraction, 201.19 g of MA and 125.80 g of SV were boiled in 4000 mL of distilled water for 30 min. The extracts were cooled and filtered. The final PA concentrations in the extracts were determined according to the method described in Section 5.2.3.

#### 5.2.3. Quantitative Analysis Using LC-HRMS

A 10 mL aliquot of the obtained weed extract was transferred into a 50 mL plastic centrifuge tube and 10 mL of a 1% formic acid solution in acetonitrile was added, followed by a 30 s vortexing at 3000 rpm. The QuEChERS salt mix, containing magnesium sulfate, sodium chloride, trisodium citrate dihydrate, and disodium hydrogen citrate sesquihydrate in the proportions of 2.0 g:0.5 g:0.5 g:0.25 g, was then added, and the tube was vortexed again for 30 s at 3000 rpm. The samples were placed in an ultrasonic bath for 15 min without additional heating, followed by centrifugation at 4000 rpm for 10 min. The acetonitrile layer was filtered through a 0.45 µm pore-size filter and analyzed using LC-HRMS.

Extracts and distilled water used for extraction were analyzed using an Agilent 1290 Infinity II HPLC system coupled with an Agilent 6530 qTOF MS (Agilent Technologies, Waldbronn, Germany). Separation was performed on a Zorbax Extend C18 Rapid Resolution HD (2.1 × 100 mm, 1.8 µm) column (Agilent Technologies, Waldbronn, Germany) at a flow rate of 0.3 mL/min. The column oven was set at 60 °C, and the sample injection volume was 0.5 µL, with a 40 s needle wash using 70% methanol. The injection sequence followed a calibration order from lowest to highest concentration, with blanks run before and after each analytical sample. A blank sample was injected at both the beginning and end of each sequence, using LC-MS-grade methanol as the blank.

The mobile phase consisted of solvent A (5 mM ammonium formate with 0.2% formic acid in deionized water) and solvent B (10 mM ammonium formate in methanol). The gradient profile was as follows: 95% A and 5% B at 0.0 min, held until 0.5 min, transitioning to 50% A and 50% B by 3.5 min, and remaining constant until 5.5 min. At 6.5 min, the composition changed to 25% A and 75% B, reaching 0% A and 100% B at 8.0 min, which was maintained until 13.0 min. The system returned to 95% A and 5% B at 14.0 min, allowing column re-equilibration, and was held at this composition until the run ended at 19.0 min.

The mass spectrometer was operated with the following settings: fragmentation voltage: 70 V, gas temperature: 325 °C, drying gas flow: 10 L min^−1^, nebulizer pressure: 25 psi, sheath gas temperature: 400 °C, and sheath gas flow: 12 L min^−1^. Electrospray ionization (ESI) was used in positive mode, with spectra acquired in the m/z range of 50–950. Internal reference masses 121.050873 *m*/*z* and 922.009798 *m*/*z* (G1969-85001 ESI-TOF Reference Mass Solution Kit, Agilent Technologies, Santa Clara, CA, USA) were used for mass calibration [67]. LC-MS data were processed using Agilent MassHunter Qualitative Analysis 10.0 software (Agilent Technologies, Santa Clara, CA, USA).

The quantitative determination of pyrrolizidine alkaloids was performed using the external standard method. Only the PAs currently monitored under EU Regulation No. 2023/915 were included in the experiment. Standard solutions for calibration curve preparation and limit of quantification (LOQ) determination were prepared in methanol within the concentration range of 5 to 100 ng mL^−1^. Each calibration point was analyzed using three replicate injections, and the mean value was used for quantification. Linear regression was performed for each calibration curve (Table 3).

### 5.3. Fate of Pyrrolizidine Alkaloids in Soil

#### 5.3.1. Soil Samples

Two types of soil were used in this study: Soil N1 was a sod-podzolic soil collected on 4 April 2024 from Priekuļi Parish, Cēsis County (57°19′11.4″ N 25°19′23.5″ E). Soil N2 was a sodium carbonate soil collected on 3 April 2024 from Pūre Parish, Abava County (57°00′40.0″ N 22°55′07.5″ E). These soil types were selected as they are among the most typical for agricultural practices in Latvia. The agrochemical properties of the soils are summarized in Table 4. Prior to the leaching analysis, the soil samples were tested for PA content using the LC-HRMS methods described in Section 5.3.3.

#### 5.3.2. Design and Settings

Plastic cylinders (6 cm in diameter) with a tapered end and a filter at the bottom were used for the experiment. Two different soil layer configurations were established: a 10 cm topsoil layer, representing the biologically active zone with high microbial and root interactions, and a 20 cm profile consisting of topsoil and subsoil to evaluate PA transport beyond the surface and potential sequestration or degradation. Based on soil composition, moisture content, and layer thickness, the maximum volume of extract (or water) required to reach 85–100% moisture saturation was determined (Supplementary Table S5). The extract volume varied depending on the soil’s saturation capacity to ensure uniform PA distribution and simulate natural soil moisture conditions. The soil analysis followed a structured protocol in which treatments were applied on day 0, when the soil was treated with extract (as detailed in Supplementary Table S5), and PA fate assessments were conducted on days 3, 5, 7, 10, and 14, during which soil samples were rinsed with the appropriate amount of water. To prevent evaporation from both the soil and the collection vessel, the system was hermetically sealed. Percolation rates were set between 10 and 60 min per 2.5 cm of soil, corresponding to 40–240 min for the 10 cm topsoil layer and 80–440 min for the 20 cm topsoil-plus-subsoil layer. These conditions simulated natural infiltration rates, ensuring that all treatments experienced realistic soil water movement. Supplementary Table S5 also presents the total water volume (mL) used for leaching each sample and its conversion to millimeters (mm). A total of 96 samples were prepared, with each treatment and soil configuration replicated to ensure statistical reliability and robustness of the results. Samples were immediately filtered and stored at −20 °C until analysis.

#### 5.3.3. Quantitative Analysis Using LC-HRMS

A 10 mL aliquot of the obtained water fraction was transferred into a 50 mL plastic centrifuge tube and 10 mL of a 1% formic acid solution in acetonitrile was added, followed by a 30 s vortex at 3000 rpm. The QuEChERS salt mix, containing magnesium sulfate and sodium chloride in a ratio of 4.0 g:1.0 g, was then added, and the tube was vortexed again for 30 s at 3000 rpm. The samples were placed in an ultrasonic bath for 15 min without additional heating, followed by centrifugation at 4000 rpm for 10 min. The acetonitrile layer was filtered through a 0.45 µm pore-size filter and analyzed using LC-HRMS according to the method described in Section 5.2.3, with the only exception being an injection volume of 5 μL.

### 5.4. Statistical Analysis

Statistical analyses were performed using R version 4.2.2. (R Core Team, 2021). Analysis of variance (ANOVA) was carried out using the *aov* function. Duncans’s post hoc analysis was performed with the *duncan.test* function from the ‘*agricolae*’ package [72] to determine the significant differences between means.

## Figures and Tables

**Table 1:** PA content in soil (µg kg^−1^) after 14 days of leaching *Myosotis arvensis* extracts.

Extract Type	Soil	Soil Layer	Hs	HsNO	ImNO
Cold extract	N1	10 cm	3.54 ± 3.54 ^a^	0.00 ± 0.00	
	N1	20 cm	1.12 ± 1.12 ^b^		
	N2	10 cm	1.84 ± 1.84 ^c^		
	N2	20 cm	3.62 ± 3.62 ^a^		
Hot extract	N1	10 cm	6.48 ± 6.48 ^de^		
	N1	20 cm	7.14 ± 7.14 ^d^		
	N2	10 cm	5.91 ± 5.91 ^e^		
	N2	20 cm	8.79 ± 8.79 ^f^		

Means marked with the same letter within each column are not significantly different at *p* ≤ 0.05 according to Duncan’s multiple range test.

**Table 2:** PA content (µg kg^−1^) in soil after 14 days of leaching *Senecio vulgaris* extracts.

Extract Type	Soil	Soil Layer	Ir	Sp	Us	IrNO	SpNO	UsNO
Cold extract	N1	10 cm	16.38 ± 1.61 ^a^	4.42 ± 0.54 ^a^	4.16 ± 0.35 ^a^	0.00 ± 0.00		0.00 ± 0.00
	N1	20 cm	26.01 ± 2.45 ^b^	9.37 ± 0.32 ^b^	6.83 ± 0.80 ^b^		0.00 ± 0.00 ^a^	
	N2	10 cm	2.37 ± 0.31 ^c^	6.50 ± 0.69 ^c^	1.39 ± 0.11 ^c^			
	N2	20 cm	6.17 ± 0.62 ^d^	1.43 ± 0.19 ^d^	2.76 ± 0.14 ^d^			
Hot extract	N1	10 cm	27.43 ± 2.19 ^b^	5.92 ± 0.61 ^c^	4.69 ± 0.36 ^a^		0.08 ± 0.01 ^b^	
	N1	20 cm	40.16 ± 2.85 ^e^	7.35 ± 0.51 ^e^	6.92 ± 1.17 ^b^		0.13 ± 0.01 ^c^	
	N2	10 cm	4.34 ± 0.09 ^cd^	0.76 ± 0.04 ^f^	1.17 ± 0.17 ^c^		0.08 ± 0.01 ^b^	
	N2	20 cm	12.42 ± 0.90 ^f^	2.21 ± 0.26 ^g^	2.92 ± 0.25 ^d^		0.13 ± 0.01 ^c^	

Means marked with the same letter within each column are not significantly different at *p* ≤ 0.05 according to Duncan’s multiple range test.

**Table 3:** LC-HRMS parameters and calibration data for the analysis of pyrrolizidine alkaloids.

PA	t_R_, min	[M+H]_t_^+^	[M+H]_p_^+^	Δ, ppm	a	b	r^2^	LOQ, ng mL^−1^	Esterification
Intermedine-N-oxide	4.70	316.1755	316.1753	−0.64	7086	−59125	0.9992	7.09	ME
Seneciphylline	5.13	334.1649	334.1653	1.20	8238	−24026	0.9987	5.00	CDE
Integerrimine	5.60	336.1805	336.1814	2.68	7671	−84340	0.9962	8.96	CDE
Seneciphylline-N-oxide	5.30	350.1598	350.1599	0.29	6297	−25866	0.9990	2.14	CDE
Usuramine	4.91	352.1755	352.1756	0.28	3421	−25668	0.9966	5.11	CDE
Integerrimine-N-oxide	5.79	352.1755	352.1760	1.42	5540	−34947	0.9988	4.39	CDE
Usuramine-N-oxide	5.06	368.1704	368.1714	2.72	2055	−14862	0.9928	9.71	CDE
Heliosupine	6.26	398.2173	398.2163	−2.51	7691	−35718	0.9930	3.59	DE
Heliosupine-N-oxide	6.77	414.2122	414.2123	0.24	6403	−12066	0.9957	5.50	DE

ME—monoester. DE—open-chain diester. CDE—cyclic diester.

**Table 4:** Agrochemical properties of sandy loam and loam soils used in this study.

Soil Properties	Soil N1	Soil N2
* pH (KCl)	4.9	7.5
* pH (H_2_O)	6.0	8.3
Organic matter, %	2.5	1.9
** P_2_O_2_, mg kg^−1^	124	159
** K_2_O, mg kg^−1^	119	139
** Mg, mg kg^−1^	82	1003
** Ca, mg kg^−1^	783	2259
B, mg kg^−1^	0.8	0.8
** Cu, mg kg^−1^	2.1	2.1
** Mn, mg kg^−1^	65.5	102.0
** Zn, mg kg^−1^	1.2	3.5
** S-SO_4_, mg kg^−1^	<1.0	2.0
** Fe, mg kg^−1^	633	571
** Na, mg kg^−1^	2.0	2.2
*** CEC, cmol (+)/kg	4.7	19.7
**** Sand, %	73	48
**** Silt, %	18	43
**** Clay, %	9	9
**** Soil Type	sandy loam	loam

* ISO 10390:2021 [68]. ** ISO 17586:2016 [69]. *** ISO/TS 22171:2023 [70]. **** ISO 17892-4:2016 [71].

## Data Availability

The original contributions presented in this study are included in the article/Supplementary Materials. Further inquiries can be directed at the corresponding author.

## Supplementary Materials

The following supporting information can be downloaded at https://www.mdpi.com/article/10.3390/toxins17070335/s1, Table S1: Analysis of variance of cumulative leaching of total pyrrolizidine alkaloids from Myosotis arvensis on days 3, 5, 7, 10, and 14 after application of extracts, as influenced by extraction type (cold versus hot), soil type, and soil layer. Table S2: Analysis of variance of pH of Myosotis arvensis in extracts (day 0) and soil leachates on days 3, 5, 7, 10, and 14 after application of extracts, as influenced by extraction type (cold versus hot), soil type, and soil layer. Table S3: Analysis of variance of cumulative leaching of total pyrrolizidine alkaloids from Senecio vulgaris on days 3, 5, 7, 10, and 14 after application of extracts, as influenced by extraction type (cold versus hot), soil type, and soil layer. Table S4: Analysis of variance of pH of Senecio vulgaris in extracts (day 0) and soil leachates on days 3, 5, 7, 10, and 14 after application of extracts, as influenced by extraction type (cold versus hot), soil type, and soil layer. Table S5: Experimental setup for PA leaching behavior in soil.
