# Supplementary material for: Fate of Pyrrolizidine Alkaloids in Soil: Insights from Myosotis arvensis L. and Senecio vulgaris L
Source: Toxins (Basel). 2025 Jul 2;17(7):335. doi: 10.3390/toxins17070335 (PMC12301031; doi:10.3390/toxins17070335)
Supplement: Supplementary file 1 [file toxins-17-00335-s001.zip › toxins-3715042-supplementary.pdf]

Fate of Pyrrolizidine Alkaloids in Soil: Insights from *Myosotis arvensis L.* and *Senecio vulgaris L.*

Ilva Nakurte*, Gundars Skudriņš and Ieva Mežaka

Table S1. Analysis of variance of cumulative leaching of total pyrrolizidine alkaloids from *Myosotis arvensis* on days 3, 5, 7, 10, and 14 after application of extracts, as influenced by extraction type (cold versus hot), soil type, and soil layer.

Table S2. Analysis of variance of pH of *Myosotis arvensis* in extracts (day 0) and soil leachates on days 3, 5, 7, 10, and 14 after application of extracts, as influenced by extraction type (cold versus hot), soil type, and soil layer.

Table S3. Analysis of variance of cumulative leaching of total pyrrolizidine alkaloids from *Senecio vulgaris* on days 3, 5, 7, 10, and 14 after application of extracts, as influenced by extraction type (cold versus hot), soil type, and soil layer.

Table S4. Analysis of variance of pH of *Senecio vulgaris* in extracts (day 0) and soil leachates on days 3, 5, 7, 10, and 14 after application of extracts, as influenced by extraction type (cold versus hot), soil type, and soil layer.

Table S5. Experimental setup for PA leaching behavior in soil.

Table S1. Analysis of variance of cumulative leaching of total pyrrolizidine alkaloids from *Myosotis arvensis* on days 3, 5, 7, 10, and 14 after application of extracts, as influenced by extraction type (cold versus hot), soil type, and soil layer.

Day	Factor	Degree of freedom	Sum of Squares	Mean Squares	F-value	P-value	
3	Extraction type	1	1132.2	1132.2	42.73	0.00	***
	Soil type	1	827.1	827.1	31.21	0.00	***
	Soil layer	1	506	506	19.1	0.00	***
	Residuals	28	741.9	26.5			
5	Extraction type	1	1080.8	1080.8	26.391	0.00	***
	Soil type	1	0.9	0.9	0.023	0.88	
	Soil layer	1	8.7	8.7	0.214	0.65	
	Residuals	28	1146.7	41			
7	Extraction type	1	1322.6	1322.6	30.73	0.00	***
	Soil type	1	20.9	20.9	0.486	0.49	
	Soil layer	1	25.7	25.7	0.596	0.45	
	Residuals	28	1205.1	43			
10	Extraction type	1	1275	1275	30.719	0.00	***
	Soil type	1	16.7	16.7	0.403	0.53	
	Soil layer	1	61.8	61.8	1.49	0.23	
	Residuals	28	1162.1	41.5			
14	Extraction type	1	1313	1313	31.627	0.00	***
	Soil type	1	9.8	9.8	0.235	0.63	
	Soil layer	1	64.5	64.5	1.553	0.22	
	Residuals	28	1162.4	41.5			

*** $p<0.001$

Table S2. Analysis of variance of pH of *Myosotis arvensis* in extracts (day 0) and soil leachates on days 3, 5, 7, 10, and 14 after application of extracts, as influenced by extraction type (cold versus hot), soil type, and soil layer.

Day	Factor	Degree of freedom	Sum of Squares	Mean Squares	F-value	P-value	
0	Extraction type	1	15.666	15.67	78.29	0.00	***
	Soil type	1	0	0	0	0.99	
	Soil layer	1	0	0	0	1.00	
	Residuals	28	5.603	0.2			
3	Extraction type	1	0.265	0.265	0.565	0.46	
	Soil type	1	4.256	4.256	9.08	0.01	**
	Soil layer	1	0.033	0.033	0.071	0.79	
	Residuals	28	13.124	0.469			
5	Extraction type	1	0.101	0.101	0.219	0.64	
	Soil type	1	10.058	10.058	21.711	0.00	***
	Soil layer	1	1.437	1.437	3.101	0.09	
	Residuals	28	12.971	0.463			
7	Extraction type	1	0.711	0.711	1.322	0.26	
	Soil type	1	0.636	0.6356	1.181	0.29	
	Soil layer	1	1.643	1.6426	3.053	0.09	
	Residuals	28	15.064	0.538			
10	Extraction type	1	0.031	0.0306	0.062	0.81	
	Soil type	1	0.97	0.9695	1.962	0.17	
	Soil layer	1	0.004	0.0038	0.008	0.93	
	Residuals	28	13.836	0.4941			
14	Extraction type	1	0.012	0.0116	0.019	0.89	
	Soil type	1	0.613	0.6133	0.981	0.33	
	Soil layer	1	0.161	0.161	0.258	0.62	
	Residuals	28	17.496	0.6248			

** $p<0.005$, *** $p<0.001$

Table S3. Analysis of variance of cumulative leaching of total pyrrolizidine alkaloids from *Senecio vulgaris* on days 3, 5, 7, 10, and 14 after application of extracts, as influenced by extraction type (cold versus hot), soil type, and soil layer.

Day	Factor	Degree of freedom	Sum of Squares	Mean Squares	F- value	P-value	
3	Extraction type	1	101	101	6.507	0.02	*
	Soil type	1	6083	6083	390.64	0.00	***
	Soil layer	1	38	38	2.465	0.13	
	Residuals	28	436	16			
5	Extraction type	1	0	0	0.045	0.83	*
	Soil type	1	18692	18692	2036.509	<2e-16	***
	Soil layer	1	0	0	0.054	0.82	
	Residuals	28	257	9			
7	Extraction type	1	54	54	5.905	0.02	*
	Soil type	1	18756	18756	2053.381	0.00	***
	Soil layer	1	2	2	0.257	0.62	
	Residuals	28	256	9			
10	Extraction type	1	46	46	4.727	0.04	*
	Soil type	1	17825	17825	1822.51	0.00	***
	Soil layer	1	4	4	0.455	0.51	
	Residuals	28	274	10			
14	Extraction type	1	47	47	4.582	0.04	*
	Soil type	1	17519	17519	1706.735	0.00	***
	Soil layer	1	3	3	0.297	0.59	
	Residuals	28	287	10			

* p<0.05, *** p<0.001

Table S4. Analysis of variance of pH of *Senecio vulgaris* in extracts (day 0) and soil leachates on days 3, 5, 7, 10, and 14 after application of extracts, as influenced by extraction type (cold versus hot), soil type, and soil layer.

Day	Factor	Degree of freedom	Sum of Squares	Mean Squares	F-value	P-value	
0	Extraction type	1	13.52	13.52	24.89	0.00	***
	Soil type	1	0	0	0	1.00	
	Soil layer	1	0	0	0	1.00	
	Residuals	28	15.21	0.543			
3	Extraction type	1	0.004	0.004	0.006	0.94	
	Soil type	1	5.225	5.225	8.592	0.01	**
	Soil layer	1	0.069	0.069	0.114	0.74	
	Residuals	28	17.026	0.608			
5	Extraction type	1	0.015	0.015	0.015	0.90	
	Soil type	1	6.23	6.23	6.017	0.02	*
	Soil layer	1	3.672	3.672	3.546	0.07	
	Residuals	28	28.992	1.035			
7	Extraction type	1	0.361	0.3613	0.397	0.53	
	Soil type	1	1.17	1.1705	1.286	0.27	
	Soil layer	1	0.16	0.1596	0.175	0.68	
	Residuals	28	25.486	0.9102			
10	Extraction type	1	0.04	0.0399	0.034	0.86	
	Soil type	1	0.41	0.4073	0.348	0.56	
	Soil layer	1	1.1	1.0989	0.94	0.34	
	Residuals	28	32.73	1.1688			
14	Extraction type	1	0.012	0.0124	0.014	0.91	
	Soil type	1	0.458	0.4584	0.504	0.48	
	Soil layer	1	0.064	0.0639	0.07	0.79	
	Residuals	28	25.485	0.9102			

* p<0.05, ** p<0.005, *** p<0.001

Table S5. Experimental setup for PA leaching behavior in soil

Soil and Depth	Extract code	pH_0	Extract V_0, mL	Total V, mL	Leaching Depth, mm
			Cold extracts		
N1 10cm	H_2O CE	5.5	65	225	79.6
N2 10cm			100	330	116.7
N1 20cm			100	390	137.9
N2 20cm			150	460	162.7
N1 10cm	MA CE	6.1	65	225	79.6
N2 10cm			100	330	116.7
N1 20cm			100	390	137.9
N2 20cm			150	460	162.7
N1 10cm	SV CE	7.5	65	225	79.6
N2 10cm			100	330	116.7
N1 20cm			100	390	137.9
N2 20cm			150	460	162.7
			Hot extracts		
N1 10cm	H_2O HE	5.5	65	225	79.6
N2 10cm			100	330	116.7
N1 20cm			100	390	137.9
N2 20cm			150	460	162.7
N1 10cm	MA HE	4.7	65	225	79.6
N2 10cm			100	330	116.7
N1 20cm			100	390	137.9
N2 20cm			150	460	162.7
N1 10cm	SV HE	6.2	65	225	79.6
N2 10cm			100	330	116.7
N1 20cm			100	390	137.9
N2 20cm			150	460	162.7

pH_0 - Initial pH value; Extract V_0, mL – initial extract volume; H_2O – water; MA - *Myosotis arvensis*; SV - *Senecio vulgaris*
